# Flavanol-Rich Cocoa Supplementation Inhibits Mitochondrial Biogenesis Triggered by Exercise

**DOI:** 10.3390/antiox11081522

**Published:** 2022-08-04

**Authors:** Jose Angel García-Merino, Beatriz de Lucas, Karen Herrera-Rocha, Diego Moreno-Pérez, Maria Gregoria Montalvo-Lominchar, Arantxa Fernández-Romero, Catalina Santiago, Margarita Pérez-Ruiz, Mar Larrosa

**Affiliations:** 1 MAS Microbiota Group, Faculty of Biomedical and Health Sciences, Universidad Europea de Madrid, Villaviciosa de Odón, 28670 Madrid, Spain; 2 Research Group on Functional Foods and Nutraceuticals, TecNM/Instituto Tecnológico de Durango, Felipe Pescador 1830 Ote., Durango 34080, Mexico; 3 Department of Education, Research and Evaluation Methods, Comillas Pontifical University, Cantoblanco, 28015 Madrid, Spain; 4 Faculty of Sport Sciences, Universidad Europea de Madrid, Villaviciosa de Odón, 28670 Madrid, Spain; 5 Department of Health and Human Performance, Faculty of Physical Activity and Sports Sciences-INEF, Polytechnic University of Madrid, 28040 Madrid, Spain; 6 Department of Nutrition and Food Science, School of Pharmacy, Complutense University of Madrid (UCM), 28040 Madrid, Spain

**Keywords:** procyanidin B2, reactive oxygen species, IL-6, ND1, CYTB, SOD, maximal aerobic capacity, exercise performance, VO_2max_

## Abstract

The potential role of cocoa supplementation in an exercise context remains unclear. We describe the effects of flavanol-rich cocoa supplementation during training on exercise performance and mitochondrial biogenesis. Forty-two male endurance athletes at the beginning of the training season received either 5 g of cocoa (425 mg of flavanols) or maltodextrin (control) daily for 10 weeks. Two different doses of cocoa (equivalent to 5 g and 15 g per day of cocoa for a 70 kg person) were tested in a mouse exercise training study. In the athletes, while both groups had improved exercise performance, the maximal aerobic speed increased only in the control group. A mitochondrial DNA analysis revealed that the control group responded to training by increasing the mitochondrial load whereas the cocoa group showed no increase. Oxidative stress was lower in the cocoa group than in the control group, together with lower interleukin-6 levels. In the muscle of mice receiving cocoa, we corroborated an inhibition of mitochondrial biogenesis, which might be mediated by the decrease in the expression of nuclear factor erythroid-2-related factor 2. Our study shows that supplementation with flavanol-rich cocoa during the training period inhibits mitochondrial biogenesis adaptation through the inhibition of reactive oxygen species generation without impacting exercise performance.

## 1. Introduction

Physical activity generates various biochemical signals that trigger exercise adaptations in almost every organ of the body [1]. One of these adaptations is mitochondrial biogenesis, which boosts the aerobic capacity of skeletal muscle and, consequently, enhances exercise performance [2]. Performance-enhancing dietary supplements are increasingly used by athletes as a means of bolstering endurance levels [3]. Accordingly, dietary supplements that enhance mitochondrial biogenesis would be anticipated to improve physical performance. One supplement that may fit this description is flavanol-rich cocoa, which has been reported to induce physiological changes similar to those induced by exercise [4]. For example, the consumption of the flavanol-rich fraction of cocoa has been reported to promote mitochondrial biogenesis in mice [5]. In addition, in vitro studies have shown that the flavonoids epicatechin and procyanidin B2, which are present in high amounts in cocoa products, have an antagonistic action on mitochondrial respiration [6] and likely have mitochondria-promoting properties. Indeed, epicatechin promotes mitochondrial biogenesis in cultured myotubes [7] and procyanidin B2 increases the mitochondrial DNA content in cultured mesangial cells under conditions of high-dose glucosamine [8]. Likewise, epicatechin administered to exercising mice increases the volume and number of mitochondria in the muscle, improving physical performance [9].

Studies in humans have indicated that sub-chronic and chronic cocoa intake reduces the oxygen cost of cycling, thereby improving exercise performance [10], and increases the maximal oxidative metabolic capacity (VO_2max_) and power output during an incremental cycle test in untrained participants [11]. Other studies, however, failed to find any improvement in VO_2max_ in active males [12], or in young soccer players [13]. Moreover, Schwarz and colleagues recently reported that epicatechin supplementation for four weeks in conjunction with cycling exercise inhibits aerobic and mitochondrial adaptations during cycle exercise training [14].

To date, there is no clear evidence for the usefulness of cocoa on the performance of endurance athletes. Furthermore, few studies have characterized the compounds contained in cocoa supplements, so it is difficult to establish which components are responsible for the observed outcomes. Our study aimed to determine whether the intake of flavanol-rich cocoa increases mitochondrial biogenesis and sports performance in athletes and in a murine exercise model.

## 2. Materials and Methods

### 2.1. Materials

Maltodextrin was purchased from Prozis (Esposende, Portugal). High-flavanol cocoa (83 mg/g) was purchased from Chococru (London, UK). The polyphenolic characterization of high-flavanol cocoa has been described previously [15]; the main constituents include procyanidin B2 (68 mg/g), (−) epicatechin (8 mg/g), catechin (3.85 mg/g), and procyanidin B1 (3.34 mg/g).

### 2.2. Experimental Design, Dietary Supplementation and Intervention Compliance Control

We designed a randomized, parallel-group, placebo-controlled trial. The research ethics committee on drugs of the Comunidad de Madrid, Spain, approved the study (reference: 07/694487.9/17). All procedures were in accordance with the 1964 Helsinki Declaration and its later amendments, and the study was registered on ClinicalTrials.gov (accessed on 22 June 2022) (ID: NCT04301583). Participants were recruited from recreational sports clubs across Madrid through visits, mail and telephone calls. A total of 56 athletes were screened for the study, of whom 54 met the following the inclusion criteria: (1) male endurance cross-country athletes, (2) 18–50 years of age, (3) high physical condition (VO_2_ ≥ 55 mL/kg min^−1^), and (4) body mass index 18–25 kg/m^2^. The exclusion criteria were as follows: (1) consumption of any kind of nutritional or ergogenic supplement, (2) smoking, (3) chronic medication, and (4) gastrointestinal surgery, or any diagnosed disease. Written informed consent was obtained from all participants.

**Figure 1:**
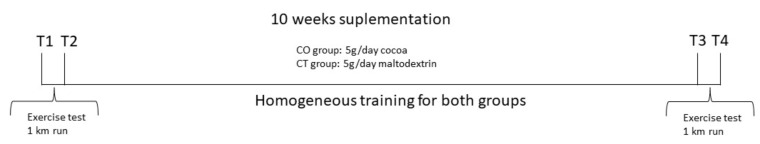
Study design.

Subjects were randomized into two equal-size treatment groups using the RAND function of Excel (Microsoft Office Excel, 2019). The study lasted 10 weeks, which was enough time for the exercise to produce physiological adaptations [16]. Athletes performed two bouts of acute exercise (exercise test + 1 km run) during their training period: one before the start of supplementation and the other after 10 weeks of supplementation. Blood samples were collected on four occasions: at the beginning of the experimental period at rest (T1), 10–15 min after finishing the exercise bout (T2), at rest after 10 weeks of supplementation (T3) and 10–15 min after the bout of acute exercise (T4) (Figure 1). The cocoa supplementation (CO) group received 5 g of fat-reduced cocoa daily that provided 425 mg of flavanols, whereas the control (CT) group received 5 g of maltodextrin. The dose of flavanols is similar to that used in other nutritional intervention studies [10,17]. Maltodextrin is used as a carbohydrate supplement for athletes; however, 5 g is insufficient to affect daily macronutrient and energy intake or sporting performance [18]. Both supplements were provided in identical single-dose paper sachets to dissolve in semi-skimmed milk. Sachets were dispensed by postal mail and researchers that collected and analyzed the data were blinded to the group assignments. Participants were called by telephone every week to check that they were taking the corresponding supplement. All measurements were recorded before and after the 10-week endurance training intervention.

### 2.3. Exercise Test and 1 km Run

Participants were individually scheduled to attend the test laboratory between 8:00 am and 12:00 am. They refrained from any physical activity 24 h before the day of the physical test. The protocol followed has been described in a previous study [19], which observed exercise-induced metabolic changes. Briefly, all participants performed a standardized 10 min warm-up of continuous running on a treadmill (H/P/Cosmos Venus, Nussdorf-Traunstein, Germany) at 60% of their maximum heart rate (HR). After the warm-up, they ran with a slope of 1% at a speed of 10 km/h, with increments of 0.3 km/h every 30 s until volitional exhaustion. Participants were verbally encouraged to give their maximal effort, particularly towards the end of the test. Fifteen min after the exercise test, the participants performed a 1 km run on an outdoor athletic track at maximum speed and the time needed to cover this distance was recorded.

### 2.4. Determination of Maximal Oxygen Uptake, First and Second Ventilatory Threshold, and Maximal Aerobic Speed

During the tests, gas exchange data were continuously collected using an automatic breath-by-breath measurement system (Ultima^TM^ Series, MGC Diagnostic Corp., St. Paul, MN, USA) that was calibrated according to the manufacturer’s instructions. The volume calibration was performed at different flow rates with a 3 L calibration syringe that allowed an error of <3%. The calibration of the gas analyzer was automatically performed by the system, using the reference values of ambient gases (16% O_2_ and 4% CO_2_). During the progressive test, HR was measured with a Polar Sport-tester (Polar Electro Oy, Kempele, Finland), and oxygen consumption was constantly monitored and associated with the speed at which the first ventilatory threshold, second ventilatory threshold, and maximal aerobic speed (VT1, VT2 and MAS, respectively) were identified, as previously described [15]. Two independent observers registered VT1 and VT2, and a third observer was consulted in the event of disagreement. The MAS was associated with the last completed 30 s stage before exhaustion [20]. The determined variables were used to calculate absolute maximal oxygen consumption (VO_2max_ABS), MAS, VT1, and VT2.

### 2.5. Sports Training Monitoring

Following the pre-screening, subjects committed to following a training intervention based on the classical 3-phase model of Skinner and McLellan [21]. Three main zones were differentiated as follows: <VT1 (at or below VT1—Zone 1); VT1–VT2 (between thresholds, precisely beyond VT1 and below VT2—Zone 2) and >VT2 (at or beyond VT2—Zone 3). Subjects followed a polarized endurance-training intensity distribution that involved significant proportions of low-intensity (Zone 1) and high-intensity (Zone 3) training, and only a small proportion of moderate-intensity (Zone 2) training. The time expended in each of the aforementioned training zones was as follows: 75–80% in Zone 1, ~5% in Zone 2, and 15–20% in Zone 3 [20]. The total training load was approximately 43–7–50% for Zones 1–2–3 [22]. Participants trained 5–6 sessions per week and controlled their training intensity by continuous HR registration during all training sessions.

### 2.6. Dietary Habits

Dietary habits were recorded at T1 and T3 using a food frequency questionnaire [23] and three 24 h dietary recalls (two weekdays and one weekend day). Data were analyzed using Dietsource 3.0 software (Novartis, Barcelona, Spain) to obtain the dietary intake of carbohydrates, protein, fat and fiber, and total energy. Subjects were asked to maintain their usual diet during the intervention period. A single 5 g dose of cocoa provided 13 kcal, with 1.2 g protein, 0.7 g carbohydrates and 0.5 g fat, whereas 5 g of maltodextrin provided 19 kcal, with 0 g protein, 4.8 g carbohydrates, and 0 g fat.

### 2.7. Oxidative Stress Markers

#### 2.7.1. Lipid Peroxidation–Thiobarbituric Acid Reactive Substances Assay

Lipid peroxidation levels were measured in plasma using a protocol based on the reaction of malondialdehyde (MDA) with thiobarbituric acid (TBA). The TBA reagent was prepared just before the assay and consisted of an equal part mixture of 0.375% TBA in water and 1 M phosphoric acid. A total of 50 µL of plasma were mixed with 450 µL of TBA reagent, and the mixture was heated at 85 °C for 60 min in a dry bath, cooled on ice for 5 min, and then centrifuged at 13,000 rpm for 2 min. Finally, 200 µL of the mixture was transferred to a black 96-well plate and the fluorescence intensity (excitation 530 nm, emission 550 nm) was measured on a Jasco FP-8300 spectrofluorometer (Tokyo, Japan). Data are expressed in terms of MDA concentration, using as a standard the MDA obtained from the hydrolysis of tetraethoxypropane (Sigma-Aldrich, St. Louis, MO, USA).

#### 2.7.2. Protein Carbonylation Assay

Protein carbonylation levels were measured in plasma as described [24]. Briefly, 10 mM 2,4-dinitrophenylhydrazine (Sigma-Aldrich) was prepared in 2 M hydrochloric acid. Then, 40 µL of the sample diluted 1:2 in water and 40 µL of 2,4-dinitrophenylhydrazine were mixed in a plate, which was incubated for 10 min at room temperature in the dark. Subsequently, 20 µL of 6 M sodium hydroxide were added to each well, and absorbance was recorded at 450 nm for 25 min in a SPECTROstar Nano spectrophotometer (BMG-LABTECH, Ortenberg, Germany). Bovine serum albumin (BSA) was used as a negative control, and oxidized BSA as a positive control, prepared as described [25]. All data were normalized to the amount of plasma protein content, which was determined for each sample using the DC protein assay (Bio-Rad Laboratories, Barcelona, Spain).

#### 2.7.3. Superoxide Dismutase Assay

Superoxide dismutase (SOD) activity was measured in plasma using a published protocol [26] involving xanthine oxidase and the water-soluble tetrazolium salt WST-1. Absorbance was registered for 8 min at 438 nm in a SPECTROstar Nano spectrophotometer (BMG-LABTECH).

### 2.8. Interleukin-6 Analysis

Plasma interleukin-6 (IL-6) levels were measured with a commercial assay (Human IL-6 High Sensitivity ELISA; Diaclone, CEDEX, Besançon, France).

### 2.9. Murine Study

A total of 60 male C57BL/6J mice (Envigo, Barcelona, Spain), 8–10 weeks old, were randomly divided into the following 6 groups (n = 10 per group): (1) untrained control group receiving standard rodent chow (CONT), (2) untrained group supplemented with cocoa at 8.2 mg/kg body weight (b.w.) (CO), (3) untrained group receiving high-dose cocoa at 24.6 mg/kg b.w (HCO), (4) control exercise-trained group (CONTEx), (5) exercise-trained group supplemented with cocoa at 8.2 mg/kg b.w. (COEx), and (6) exercise-trained group supplemented with high-dose cocoa at 24.6 mg/kg b.w (HCOEx). Cocoa doses were equivalent to the daily intake of 5 g or 15 g of cocoa for a 70 kg person according to an allometric scale [27]. The cocoa was the same as that used in the human study and was mixed into pellets at 8.2 g/kg and 24.6 g/kg. The chow diet provided an approximate daily intake of 25 mg and 75 mg of cocoa (considering that they consumed ca. 3 g of chow per day). The mice were kept on a 12:12 h light/dark cycle and had ad libitum access to water and food. The intervention lasted for 4 weeks, and body weight and food intake were monitored 3 times per week throughout the experiment. At the end of the study, mice were anesthetized with sodium pentobarbital and were exsanguinated by cardiac puncture. The quadriceps muscle of the right limb was then excised and quickly frozen in liquid nitrogen and stored at −80 °C for further processing. All procedures were approved by the Ethical Committee for animal experimentation of Comunidad de Madrid (PROEX 093/17).

#### Mice Training Protocol

The exercise protocol was established according to the previous protocol [28], in which other antioxidants were tested, and according to a pilot study that we previously performed, in which we established that 5 days of exercise produced an increment in mitochondrial biogenesis. The exercise groups (CONTEx, COEx and HCOEx) were first acclimatized to the treadmill (Rodent Treadmill NG, Ugo Basile, Gemonio, Italy) by daily running sessions at 10 m/min for 5 consecutive days. Because the mice did not have the same predisposition to run, an incremental test was individually performed for each mouse to adapt the exercise conditions to each animal. The incremental test started with a 3 min warm-up at 5 m/min followed by increments of 1 m/min every minute until animals refused to run, at which point the treadmill speed was reduced to 10 m/min for 1 min and then to 5 m/min for 3 min. For the subsequent 4 days, mice started the warm-up at 5 m/min for 3 min, and afterwards they ran at 85% of their maximum running velocity until they refused to run. Refusal to run was indicated when they touched the end of the treadmill three times. The treadmill grade was permanently set at 0%. Non-exercised groups were exposed to the same handling and room conditions as the exercised groups.

### 2.10. DNA Extraction from Human Blood Cells and Mouse Quadriceps Tissue

Drawn blood samples were collected in Vacutainer^®^ EDTA tubes and stored at −80 °C until use. For DNA extraction, 200 µL of whole blood were treated with proteinase K (Novagen^®^, Darmstadt, Germany), and DNA was then extracted using phenol/chloroform/isoamyl alcohol (24:24:1) and precipitated with 3 M sodium acetate (pH 5.2) and isopropanol. Finally, the pellets were dissolved in sterile, nuclease-free water. Quadriceps tissue samples were extracted with Trizol^TM^ Reagent (ThermoFisher Scientific, Waltham, MA, USA). DNA concentration and purity were evaluated by using the absorbance ratios 260/280 nm and 260/230 nm on a Nanodrop 2000 spectrophotometer (ThermoFisher Scientific).

### 2.11. Peroxisome Proliferator-Activated Receptor-Gamma Coactivator 1 Alpha (PGC1-α) Genotyping

We performed allelic discrimination analysis for the *PPARGC1A* Gly482Ser (rs8192678) polymorphism using a pre-designed TaqMan^®^ SNP genotyping test (ID: C_1643192_20). Amplification was carried out using the StepOne™ real-time PCR system (Life Technologies, Foster City, CA, USA) with a denaturation stage at 95 °C for 10 min, 50 denaturation cycles at 92 °C for 15 s, anneal/extension at 60 °C for 1 min and a final 30 s extension stage at 60 °C.

### 2.12. Mitochondrial DNA Copy Number

Mitochondrial DNA (mtDNA) content in human samples was assessed using quantitative real-time PCR by measuring the threshold cycle ratio of the mitochondria-encoded gene cytochrome c oxidase I *COX1* and the β-globin *HBB* gene (see primers in Table 1) in a StepOnePlus™ Thermocycler (ThermoFisher) using the PowerUp SYBR Green Master Mix (ThermoFisher) and 50 ng of genomic DNA. Amplification conditions were as follows: 50 °C for 2 min, 95 °C for 2 min, 95 °C for 15 s, 58 °C for 15 s (40 cycles), and 72 °C for 1 min. Melting curve analysis verified a single PCR product.

The mtDNA copy number in mouse muscle samples was determined according to a published protocol [29], using the Biorad CFX Connect™ real-time PCR detection system and the TB Green Premix Ex Taq II (Tli RNaseH Plus; TAKARA Bio. Inc., Kusatsu, Japan). Reactions of 10 μL contained 50 ng of DNA. Amplification conditions were 95°C for 30 s followed by 40 cycles of 95 °C for 5 s and 60 °C for 30 s. Primers and concentrations used were those described for *Nd1* and *CytB* mitochondrial genes and the constitutive ribosomal gene *18S* (Table 1). The ΔΔCt method was used to calculate the mtDNA copy number. All samples were at least performed in triplicate.

### 2.13. Nuclear Factor E2-Related Factor 2 Expression

Changes in the expression of the mitochondrial regulatory gene nuclear factor E2-related factor 2 (*Nfr2*) were determined by real-time RT-PCR. Total RNA was extracted from quadriceps muscle using the E.Z.N.A.^®^ Total RNA Kit (Omega Bio-Tek, Norcross, GA, USA) and its concentration was quantified in a NanoDrop spectrophotometer (ThermoFisher Scientific). RNA was reverse transcribed to cDNA using the PrimeScript^TM^ RT Master Mix (TAKARA Bio. Inc.). Quantitative real-time PCR was performed using a SYBER^®^ Green PCR Master Mix (Premix Ex Taq^TM^, TAKARA Bio. Inc.) on the CFX96 Touch Real-Time PCR Detection System (Bio-Rad Laboratories, Richmond, CA, USA). Thermal cycling parameters were as follows: 95 °C for 30 s, then 40 cycles of 95 °C for 5 s and the primer-specific annealing temperature (Table 1) for 30 s (60 °C). The last step was the melting curve analysis. The fold change in gene expression was calculated using the ΔΔCt method using the β-actin housekeeping gene as an internal control. All primer sequences are listed in Table 1.

### 2.14. Sample Size Calculation and Statistical Analysis

The sample size for the human study was calculated using the G*Power 3.1.9.4 software [35] and the published data of a study by Jonvick and colleagues [36] with a population similar to ours. Considering the conditions of the study, we expected to achieve a 10% increase in VO_2max_ after the intervention. Using a confidence level α of 0.05 and a power 1-β of 0.8, we determined that 21 participants per group would be needed to achieve statistical significance in the main variable.

Statistical analysis was conducted using SPSS 27.0 software (SPSS, Chicago, IL, USA). The distribution was checked for normality (Kolmogorov–Smirnov test) before analysis. Student’s t-test was applied when the data distribution was assumed to be normal. When more than two groups were compared differences were assessed by ANOVA with the Tukey-Kramer correction. The Wilcoxon range test, the Kruskal–Wallis test, or the Mann–Whitney test was used when the distribution could not be assumed to be normal. The χ^2^ test was used for comparing the frequency of alleles between groups. Spearman’s correlation analysis was used to study the relationship between variables, and linear regression analysis was performed to study the variables that predicted the mitochondria copy number. A stepwise backward-elimination method was used with the covariates that were significantly different between groups. Significance was established at *p* < 0.05. Graphs were constructed with SigmaPlot V14.5 (Systat Software, Inc., San Jose, CA, USA).

## 3. Results

### 3.1. Subject Characteristics and Genotype

**Figure 2:**
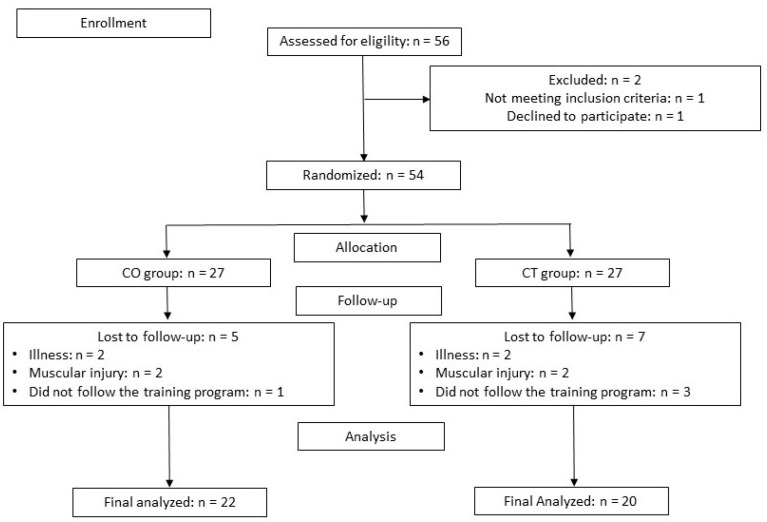
Participants flow chart. CO: cocoa group; CT: control group.

A total of 56 participants were enrolled in the present study, of which 42 completed the study: 20 in the CT group and 22 in the CO group. Seven subjects in the CT group and 5 in the CO group did not complete the study because of muscle injury or because they did not follow the training program (Figure 2).

No significant differences were found in the age, body weight, height, or body mass index between groups (Table 2).

The transcriptional coactivator PGC-1α is a key factor in mediating exercise-training-induced adaptations [37], and its genetic variants are associated with endurance capacity [38]. For this reason, all of the subjects were genotyped for the Gly482Ser polymorphism. The frequencies of the genotypes for the homozygous major allele (CC), heterozygous allele (CT), and homozygous minor allele (TT) were similar in both groups, with no significant differences (χ^2^ = 2.54; *p* = 0.281) (Table 3).

### 3.2. Dietary Habits

The analysis of the energy, macronutrient and fiber data obtained from the food frequency questionnaire and the three 24 h recalls revealed no significant intergroup differences in T1 and T2 (Table 4), and both assessment methods indicated that nutritional intake remained constant throughout the study (Table 4). Carbohydrate intake was in the lower range of the European Food Safety Authority (EFSA) recommendations of intake of 45–60% of total energy intake [39]. Fiber intake was adequate (above the recommended 25 g/day), and protein intake was within the recommendations for endurance athletes of the American Dietetic Association and the American College of Sports (both recommend an intake of 1.2–2 g/kg body mass/day) [40]. Fat intake approached the upper level of the EFSA recommendations (range 20–35% of total energy intake).

### 3.3. Exercise Performance

Both groups showed improvements in VO_2max_, VT1 and VT2 after the 10 weeks of training (Table 5); however, the CT group also showed a significant improvement in MAS (*p* < 0.001), whereas the CO group did not (*p* = 0.572). No significant differences were found between the CT and CO groups at either T1 or T3 in the intergroup analysis. These results indicate that cocoa treatment had no overall effect on performance parameters, but it seemed to affect the aerobic capacity of the CO group.

### 3.4. Mitochondrial Biogenesis

**Figure 3:**
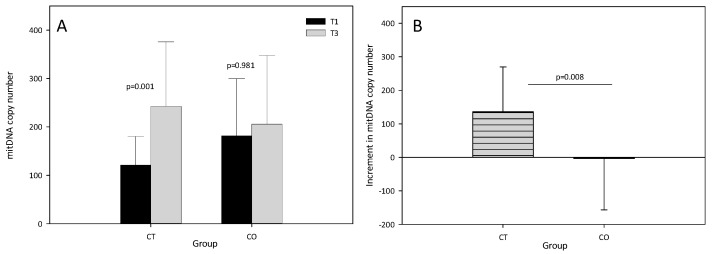
(**A**) Mitochondrial DNA copy number at T1 and T3 in control (CT) and cocoa (CO) groups; (**B**) Increment (T3–T1) in mitochondrial DNA copy number in CT and CO groups. The values represent the mean ± SD.

The results showed that the mtDNA copy number significantly increased in the CT group after the training period (T1 = 120.94 ± 59.38, T3 = 242.66 ± 133.14, *p* = 0.001). By contrast, no increase in the mtDNA copy number was found in the CO group (T1 = 181.21 ± 118.21, T3 = 205.88 ± 141.57, *p* = 0.981) (Figure 3A), suggesting that mitochondrial biogenesis was inhibited. The intergroup analysis revealed a significant difference in the increase in the mtDNA copy number between T1 and T3 (Figure 3B).

**Figure 4:**
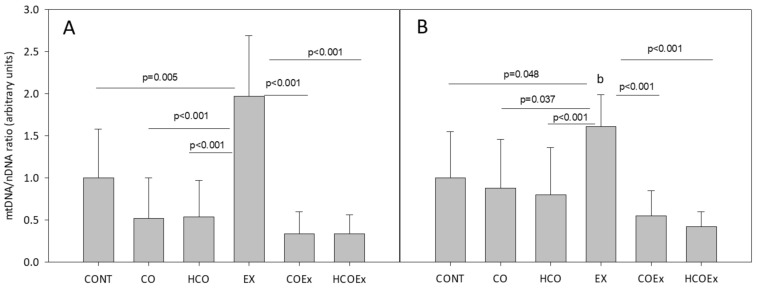
Analysis of mitochondrial/nuclear gene ratio for *Cytb* (**A**) and *Nd1* (**B**). Values represent the mean ± SD. CONT: untrained control group receiving standard rodent chow; CO: untrained group supplemented with cocoa at 8.2 mg/kg body weight (b.w.); HCO: untrained group receiving high-dose cocoa at 24.6 mg/kg b.w.; CONTEx: control exercise-trained group; COEx: exercise-trained group supplemented with cocoa at 8.2 mg/kg; HCOEx: exercise-trained group supplemented with high-dose cocoa at 24.6 mg/kg b.w.

We also studied the effect of cocoa supplementation on mitochondrial biogenesis in the experimental mouse study, which revealed that the level of the mitochondrial genes *Cytb* (*p* = 0.005; Figure 4A) and *Nd1* (*p* = 0.048; Figure 4B) significantly increased after exercise training (EX group vs. CONT group). However, in those mice that performed exercise and received flavanol-rich cocoa (COEx group vs. HCOEx group), the evident increase in mtDNA did not occur (Figure 4A,B). The apparent inhibition of mitochondrial biogenesis occurred to a similar extent at both doses of cocoa, indicating that the lowest dose was sufficient to exert the observed effect. Mouse groups that did not exercise showed no difference in mtDNA content when supplemented or not with cocoa (Figure 4).

### 3.5. Oxidative Stress Markers

Physical exercise is known to increases oxidative stress, principally in the form of reactive oxygen species (ROS), which serve as signals to trigger exercise adaptations [41] and promote mitochondrial biogenesis [42]. As cocoa has previously been shown to have antioxidant properties [4], we evaluated the effect of cocoa supplementation on different markers of lipid peroxidation (by measuring thiobarbituric acid reactive substances, TBARS), protein peroxidation (by measuring protein carbonyl derivatives), and antioxidant SOD activity, in the plasma of athletes.

**Figure 5:**
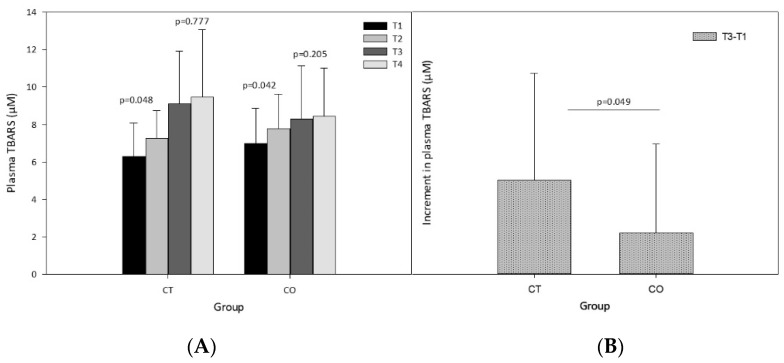
Plasma thiobarbituric acid reactive substances (TBARS). (**A**) TBARS levels at T1, T2, T3 and T4 in control (CT) and cocoa (CO) groups; (**B**) Chronic increment (T3–T1) in plasma TBARS levels in both groups. Values represent the mean ± SD.

The analysis of plasma lipid peroxidation revealed a significant increase after the exercise sessions and after 10 weeks of exercise training (Figure 5A). Before beginning the intervention (supplementation and exercise) the exercise session (T1 vs. T2) significantly increased the levels of TBARS in both groups (Figure 5A; CT group *p* = 0.048, CO group *p* = 0.042). The same analysis after 10 weeks of supplementation and training revealed that the exercise bout did not significantly increase TBARS levels (T3 vs. T4) (Figure 5A; CT group *p* = 0.777, CO group *p* = 0.205), likely due to the period of adaptation to the exercise provided by the training over the 10 weeks. Moreover, when we assessed the increase in the basal levels of TBARS initially and after the 10-week exercise and supplementation period, we found that the increase was significantly less pronounced in the CO group than in the CT group (Figure 5B).

**Figure 6:**
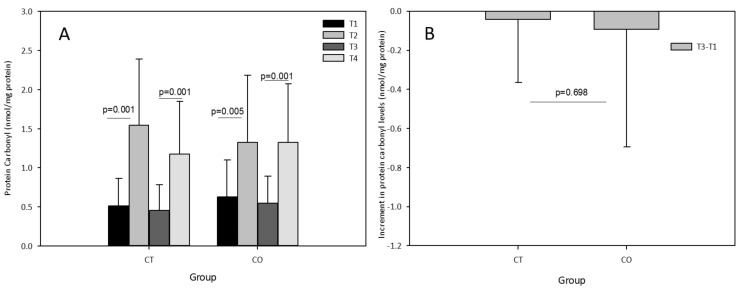
(**A**) Protein carbonyl levels in plasma of control (CT) and cocoa (CO) groups in T1, T2, T3 and T4. (**B**) Chronic increment (T3–T1) in protein carbonyl plasma levels in both groups. Values represent the mean ± SD.

The acute exercise sessions also significantly increased the levels of carbonyl proteins in both groups, initially and after the 10-week training and supplementation period (Figure 6A). No effect of cocoa supplementation was observed after the acute exercise session at the end of the training period (T3–T4) with an increase similar to that initially detected (T2–T1) in the CO group, and no chronic effect was detected (T3–T1) (Figure 6B).

**Figure 7:**
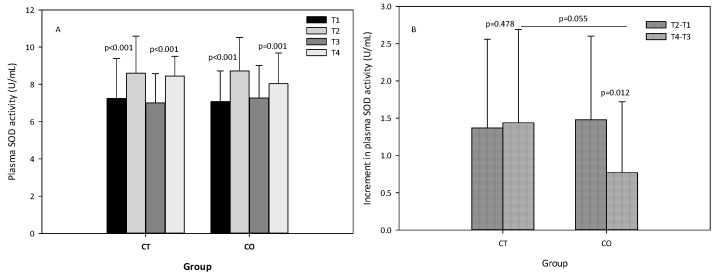
(**A**) plasma superoxide dismutase (SOD) levels of control (CT) and cocoa (CO) groups in T1, T2, T3 and T4. (**B**) Increments in SOD levels due to a bout of exercise before (T2–T1) and after 10 weeks of cocoa supplementation (T4–T3) in CT and CO groups. Values represent the mean ± SD.

The analysis of SOD activity revealed that, as expected, the exercise bout initially produced an increase in SOD activity, and after the 10 weeks of intervention (Figure 7A). When the increases initially and after the 10 weeks of supplementation were compared between groups, we observed that the increase in SOD activity was attenuated in the second bout of exercise (negative values) and less pronounced in the CO group (*p* = 0.012) than in the CT group (*p* = 0.478) (Figure 7B), suggesting that cocoa consumption influences SOD activity. When both increases (T4–T3) were compared, the difference between the two groups was almost significant (*p* = 0.055) (Figure 7B).

### 3.6. Interleukin-6 Plasma Levels

**Figure 8:**
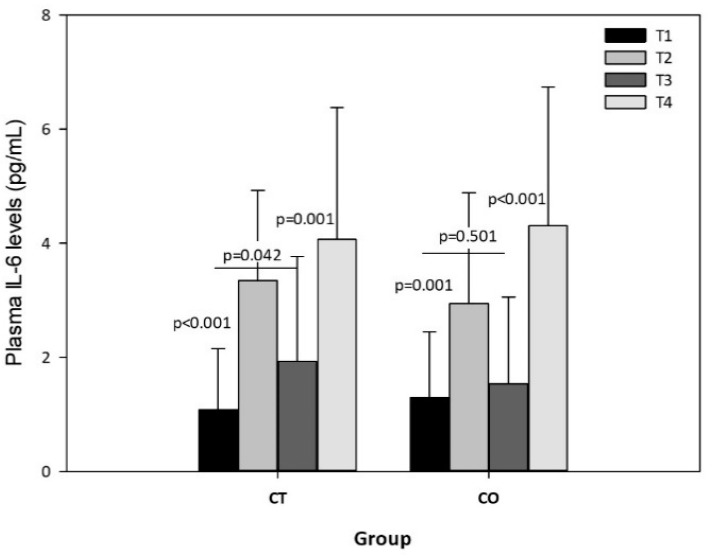
Plasma IL-6 levels in CT and CO groups in T1, T2, T3 and T4. The values represent the mean ± SD.

The exercise session produced significant increases in plasma IL-6 levels in both groups, both before and after the 10-week intervention (Figure 8), but no significant differences were observed in the intergroup analysis, indicating that chronic consumption of cocoa has no effect on the acute release of IL-6 due to exercise. Nevertheless, the intragroup analysis of IL-6 in rest conditions (T1 vs. T3) revealed an increase in the CT group over the 10 weeks (*p* = 0.042) but not in the CO group (*p* = 0.501) (Figure 8).

### 3.7. Nuclear Factor Erythroid-Derived 2-like 2 Expression

**Figure 9:**
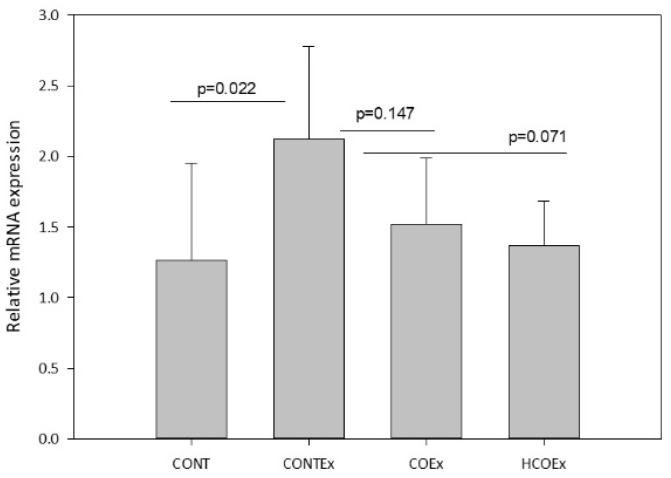
*Nfr2* gene expression in the quadriceps femoral muscle of control mice (CONT) and mice that performed exercise (CONTEx) and were fed with chow containing 8.2 g/kg (COEx) and 24.6 g/kg (HCOEx). Values represent the mean ± SD.

Regular exercise produces ROS that, in turn, promote mitochondrial biogenesis through *Nfr2*, a redox-sensitive transcriptional regulator that is required for mitochondrial biogenesis and for the antioxidant transcriptional response to acute exercise and exercise training [43]. We determined the expression of *Nfr2* in the quadriceps femoral muscle of mice, finding that exercise induced an increase in its expression in the CONTEx group in comparison with the CONT group (Figure 9). Both cocoa supplementation doses appeared to lower *Nfr2* expression in a dose-dependent manner; however, the reductions were not significant (Figure 9).

### 3.8. Analysis of the Relationship between the Variables of the Human Intervention Study

We performed correlation and linear regression analyses to better understand the relationship between mitochondrial biogenesis and the other variables. The correlation results showed a statistically positive rho = 0.477 and a significant (*p* = 0.002) correlation between the increase in the mtDNA copy number and the chronic increase in plasma MDA levels. The linear regression analysis confirmed that the increase in the mtDNA copy number was dependent on the increase in plasma MDA levels (R = 0.353 (*p* = 0.027)). The regression indicated that the predictors of performance (t1km) are relative to the VO_2_ (β = −0.401: *p* = 0.07) and the IL-6 levels at T3 (β = −0.373; *p* = 0.013), but not to the mtDNA copy number.

## 4. Discussion

Regular physical exercise leads to a series of physiological adaptations that improve athletic performance [1], and this can be enhanced by the use of dietary supplements [3]. Whether cocoa supplementation improves physical performance remains controversial, with conflicting results in the literature. For example, some studies indicate that cocoa or its major flavanols may increase performance, aerobic capacity or mitochondrial biogenesis, whereas other studies suggest that it has no effect or even a negative impact [5,6,7,9,14]. Given this uncertainty, we designed a study of endurance athletes that were randomized at the beginning of their training season to receive either flavanol-rich cocoa or a control maltodextrin supplement for 10 weeks. We also used a murine model to study the changes at the muscle level in more detail. Mice were exercise-trained and supplemented with cocoa at a dose equivalent to that used in the athlete study and at a dose 3-fold higher.

Our results showed that exercise training improved the VO_2max_ and the ventilatory thresholds VT1 and VT2 in both groups of athletes. However, whereas the control group also showed improvements in maximal aerobic capacity, the cocoa-supplemented group did not.

The performance of endurance exercise triggers the activation of numerous biochemical signals, including ROS, that converge on the activation of transcription factors that regulate the expression of mitochondrial-related genes, ultimately leading to an increase in mitochondrial biogenesis [44]. The effects of ROS on mitochondrial biogenesis are, however, contentious, as the response can be either an increase or a decrease in mitochondrial biogenesis depending on the strength of the stimulus and the cell type studied [45]. While ROS production was initially thought to be deleterious, there is mounting evidence that many of the oxidant and inflammatory processes that occur after acute exercise may be vital for the long-term adaptive responses to exercise training [46]. In this sense, it is possible that the dose of cocoa polyphenols (around 425 mg flavanols) administered chronically during the training adaptations to exercise in our study dampened oxidative stress, and thus the signaling for mitochondrial biogenesis. The performance of an exercise bout led to an elevation in oxidative stress, as supported by the TBARS and SOD data, and this was attenuated after 10 weeks in the both CT and CO groups by the practice of physical exercise and the increased physical condition, as expected [47], but this was more marked in athletes supplemented with cocoa, which may diminish the adaptations to exercise [48,49]. The antioxidant activity of cocoa and its main compounds such as catechins are well known [50,51,52]. Epicatechin and procyanidin B2 have direct and indirect effects on mitochondrial function by quenching ROS production, preserving membrane integrity and increasing ATP production, and they are possibly beneficial for diseases associated with high oxidative stress [53]. The use of supplements with antioxidant activity in athletes is provocative. A typical approach in many studies is the utilization of food supplements that suppress ROS production induced by exercise, to attenuate muscle damage [54,55,56]. That being said, the use of antioxidants such as vitamin C has been shown to delay training adaptations in skeletal muscle including the induction of PGC-1α expression and mitochondrial biogenesis [57,58]. A recent meta-analysis concluded that the use of vitamin C or E does not impede improvements in maximal aerobic capacity or endurance performance [59]. Similarly, in the present study, endurance performance was unaffected by cocoa supplementation; however, at the molecular level we observed an inhibition of mitochondrial biogenesis. One explanation for this phenomenon could be that the increase in aerobic capacity and endurance performance depends on many other factors such as calcium-mediated signaling, AMP-activated protein kinase (AMPK) activation, vascular remodeling induced by the hypoxia inducible factor-alpha and vascular endothelial growth factor, muscle energy metabolism, or the athlete’s genetic background, which might trigger different levels of response in terms of adaptations with the same exercise program [60]. We, however, discarded genetic variants of PGC-1α as a major genetic factor in mediating exercise-training-induced adaptations [37].

The reduction in ROS levels by cocoa supplementation in our study might be mediated by the transcription factor *Nrf2*. We observed a trend for a decrease in the expression levels of *Nrf2* in the cocoa-supplemented animal model, which were significantly increased by exercise. *Nrf2* is key in the coordination of redox mechanisms triggered by exercise [61], and is involved in the expression of antioxidant enzymes such as SOD [62]. Although it was not possible to measure the expression of *Nrf2* in our athlete study, we did observe a decrease in systemic SOD activity, which supports the idea that cocoa intake modifies the expression of *Nrf2*. In keeping with this idea, it has been observed that cocoa monomers and, specifically, procyanidin B2, can activate *Nrf2* and the downstream expression of genes related to oxidative stress [63,64].

Another factor that could influence the apparent inhibition of mitochondrial biogenesis is the diet of the athletes. In our case, the diet was already rich in foods that provide a large number of compounds with antioxidant activity (vitamins, polyphenols, etc.) such as fruits and vegetables (data not shown), so supplementation with cocoa may have provided an extra amount of antioxidants that was not necessary. Cell signaling depends on the levels of ROS in the organism, so a balance between the levels of oxidative and antioxidant species must be maintained. However, finding this balance is very complex, as the biological dose–response curve in oxidative stress is unpredictable and depends on many factors that must be considered, including age, genetics, health status, and level of physical training, among others [65], so it becomes necessary to move towards a personalized diet and/or supplementation in which the use of supplements is advised depending on the individual characteristics of the population group or subject. It should also be taken into account that the cocoa used in the present study was a cocoa rich in procyanidins with a medium-low degree of polymerization [15], which could have enhanced its absorption and observed effects [66].

We also observed that the levels of IL-6 at rest increased after 10 weeks of training in the CT group but not in the cocoa-supplemented group supplementation. Exercise causes an immediate release of IL-6 from the muscle that increases lipolysis and energy uptake in the muscle [67], and augments mitochondria density, improving muscle efficiency [16]. In addition, immune-cell-derived IL-6 is elevated by exercise, which repairs skeletal muscle damage and enhances muscle regeneration [68]. If the acute increase is not accompanied by a period of rest and adequate nutrition, then the effect can, however, become detrimental, leading to impaired muscle function [68]. In this sense, the use of dietary supplements has been shown to decrease both acute and chronic IL-6 increases, with the inhibition of chronic IL-6 release related to beneficial effects such as the reduction in muscle injury and the adaptive response to exercise [69]. Indeed, previous studies have shown that dark chocolate consumption decreases muscle damage biomarkers [70,71]. Considering the above, cocoa supplementation in our study could be beneficial by improving adaptations, as no decrease in acute exercise performance was observed, but it prevents IL-6 levels from increasing with continued exercise.

## 5. Study Limitations

The current study had some limitations that warrant discussion. A chronic inhibition of mitochondrial biogenesis was observed in athletes receiving cocoa during the training period. Our study design did not allow us to establish whether supplementation for shorter periods or at different times (e.g., just prior to exercise) would influence these findings. The cocoa used in the present study had a high content of flavonoids that are not found in standard cocoa supplements, so the values cannot be extrapolated to other commercial cocoa powders. The study of the athletes used a single dose of cocoa, and it might have been interesting to test multiple doses to investigate dose–response effects. In addition, we did not study the bioavailability of cocoa or the plasma metabolite profile, which might be an important factor given the different bioavailability of the bioactive compounds present in cocoa. Indeed, an analysis of the plasma metabolite profile would have allowed us to test for potential links between cocoa metabolites and the observed effects. The measurement of lipid oxidation was carried out by using the TBARS method since previous studies showed the inhibition of this parameter by cocoa compounds. However, the TBARS assay is too unspecific and can involve several artifacts; therefore, other markers of lipid oxidation should be used to confirm our lipid-peroxidation-related results. Finally, the inhibition of mitochondrial biogenesis was established in blood cells of athletes and not in the muscle (although muscle was examined in the animal study), and the mechanism of inhibition should be investigated in future studies.

## 6. Conclusions

Chronic cocoa supplementation during the training period of endurance athletes inhibits mitochondrial biogenesis by decreasing oxidative stress levels, without impacting aerobic capacity or exercise performance. At the same time, cocoa prevents the long-term increase in IL-6 that results from continued exercise, which might be protective against muscle damage. The period in which the supplementation occurred, during which time great adaptations to exercise were taking place, the framework of the athletes’ diets, which were rich in fruits and vegetables, and the type of cocoa used in the study, could be determining factors in the observed effects of cocoa on athletes. For this reason, future studies are needed that consider all of these factors and others such as the type of exercise. Nutritional supplement recommendations should follow personalized strategies and be carefully tailored to well-characterized population groups.

## Figures and Tables

**Table 1:** Primer sequences and conditions.

Gene Name	Gene Accession Number	Forward	Reverse	Annealing Temperature	Primer Concentration	Reference
MT-CO1	4512 (NC_012920.1)	CGCCGACCGTTGACTATTCT	CACTATAGCAGATGCGAGCAGG	58 °C	625 nM	This study
HBB	3043 (NC_000011.10)	GAAGAGCCAAGGACAGGTAC	CAACTTCATCCACGTTCACC	60 °C	500 nM	[29]
MT-ND1	4535 (NC_012920.1)	CTAGCAGAAACAAACCGGGC	CCGGCTGCGTATTCTACGTT	60 °C	200 nM	[30]
MT-CYT	19893556 (NC_012920.1)	ATTCCTTCATGTCGGACGAG	ACTGAGAAGCCCCCTCAAAT	60 °C	200 nM	[31]
NFE2L2	4780 (NC_000002.12)	AGCACATCCAGACAGACACCAGT	TTCAGCGTGGCTGGGGATAT	60 °C	400 nM	[32]
ACTB	60 (NC_000007.14)	GGCTGTATTCCCCTCCATCG	CCAGTTGGTAACAATGCCATGT	60 °C	200 nM	[33]
Rn18S	19791 (NC_000083.5)	GCAATTATTCCCCATGAAC	GGCCTCACTAAACCATCCAA	55 °C	200 nM	[34]

**Table 2:** Baseline characteristics of the participants.

Parameters	CT (n = 20)	CO (n = 22)	*p*
Age (years)	36.45 ± 9.03	35.18 ± 7.13	0.615
Body weight (kg)	70.16 ± 8.70	71.98 ± 7.90	0.481
Height (cm)	176.20 ± 6.30	177.13 ± 5.84	0.620
BMI	22.56 ± 2.18	22.92 ± 2.09	0.582

Values are means ± standard deviation. CO: cocoa group; CT: control group; BMI: body mass index.

**Table 3:** *PPARD1A* rs8192678 genotype frequencies.

*PPARD1A* rs8192678 Allele		CT (n = 20)	CO (n = 22)	Total
CC	Observed cases	11	11	22
	% of total	26.8	26.8	53.7
CT	Observed cases	7	6	13
	% of total	17.1	14.6	31.7
TT	Observed cases	2	5	7
	% of total	2.4	12.2	14.6
Total	Observed cases	20	22	42
	% of total	46.3	53.7	100.0

**Table 4:** Dietary habits.

	CT (n = 20)			CO (n = 22)				
	T1	T3	*p*	T1	T3	*p*	*p* *	*p* **
Energy (kcal)	2402 ± 499	2051 ± 623	0.200	2093 ± 609	2162 ± 590	0.669	0.353	0.600
Carbohydrates (%E)	46.8 ± 7.3	45.6 ± 9.1	0.546	43.9 ± 6.9	43.1± 10.18	0.699	0.226	0.428
Protein (%E)	18.7 ± 2.7	19.9 ± 3.8	0.112	20.3 ± 3.9	20.58 ± 4.0	0.667	0.163	0.419
Fat (%E)	35.0 ± 7.7	34.5 ± 8.8	0.802	35.8 ± 6.2	34.7 ± 7.5	0.346	0.878	0.929
Carbohydrates (g/kg b.m)	3.7 ± 1.6	3.15 ± 1.0	0.075	3.0 ± 1.0	2.9 ± 1.1	0.581	0.187	0.326
Protein (g/kg b.m)	1.5 ± 0.9	1.4 ± 0.5	0.425	1.5 ± 0.4	1.5 ± 0.4	0.881	0.528	0.788
Fat (g/kg b.m)	1.2 ± 0.5	1.0 ± 0.41	0.248	1.2 ± 0.5	1.08 ± 0.4	0.245	0.624	0.967
Fiber (g)	33.3 ± 18.2	29.1 ± 12.2	0.146	26.4 ± 13.5	29.3 ± 16.0	0.236	0.150	0.977

Values are means ± standard deviation. CO: cocoa group; CT: control group; b.m.: body mass; E%: percentage of energy intake; *p*: intragroup comparison *p* *: intergroup comparison at T1; *p*-value **: intergroup comparison at T3.

**Table 5:** Performance variables.

	CT (n = 20)			CO (n = 22)				
	T1	T3	*p*	T1	T3	*p*	*p* *	*p* **
VO_2max_ (mL/kg/min)	57.72 ± 5.10	59.89 ± 3.59	0.032 *	59.70 ± 5.13	61.30 ± 4.79	0.038 *	0.217	0.302
VT1 (km/h)	13.18 ± 0.96	13.72 ± 1.00	0.001 *	13.6 ± 0.87	13.86 ± 0.91	0.010 *	0.138	0.640
VT2 (km/h)	15.51 ± 1.13	16.01 ± 1.29	0.001 *	16.04 ± 0.79	16.42 ± 0.98	0.010 *	0.084	0.253
MAS (km/h)	17.59 ± 1.37	18.06 ± 1.43	0.001 *	18.10 ± 1.08	17.85 ± 2.08	0.572	0.185	0.681
T1km (min)	3.24 ± 0.29	3.18 ± 0.26	0.207	3.21 ± 0.24	3.17 ± 0.24	0.207	0.692	0.915

VO_2max_: maximum oxygen consumption; MAS: maximal aerobic speed; VT1: first ventilatory threshold; VT2: second ventilator threshold; t1km: time to run 1 km; CT: control group; CO: cocoa group; *p*: intragroup comparison; *p* *: intergroup comparison at T1; *p* **: intergroup comparison at T3; *: significantly different.

## Data Availability

Data is contained within the article.
